# Supplementary material for: Determinants of advanced age pregnancy in Ethiopian; multi-level analysis of Ethiopian demographic health survey 2016
Source: PLoS One. 2024 Jun 25;19(6):e0304954. doi: 10.1371/journal.pone.0304954 (PMC11198901; doi:10.1371/journal.pone.0304954)
Supplement: S1 File — (DOC) [file pone.0304954.s001.doc]

Table 1: individual and household level characteristics of pregnant women in Ethiopian 2016 DHS (N^a^ = *3,292)*

| Individual level variables | Categories | Weighted | |
| --- | --- | --- | --- |
|  |  | Frequency | Percentage |
| Religion | Muslim | 1,534 | 46.58 |
|  | Orthodox | 692 | 29.21 |
|  | Protestant | 717 | 21.78 |
|  | Catholic and other traditional | 80 | 2.44 |
| Marital status of | Currently in union | 3,251 | 98.74 |
|  | Currently not in union | 41 | 1.26 |
| Age at first marriage (n=3,290) | Mean and SD 16.66±0.065 | 95% CI:(16.54-16.79) | |
| Age at first sex (n=3292) | Mean and SD 16.32±0.051 | 95% CI:(16.22-16.42) | |
| Mothers education level | No education | 2,389 | 72.57 |
|  | Primary | 785 | 23.83 |
|  | Secondary and above | 188 | 3.60 |
| Partner's level of education | No education | 1,698 | 52.19 |
|  | Primary | 1,224 | 37.61 |
|  | Secondary and above | 332 | 10.20 |
| Women occupation | Have no work | 1,783 | 54.16 |
|  | Have any types of work | 1,509 | 45.84 |
| Partner occupation | Agricultural works | 2,280 | 69.74 |
|  | Employed | 733 | 22.41 |
|  | Have no work | 257 | 7.86 |
| Listening Radio or watching TV | Not at all | 2,378 | 73.49 |
|  | Less than once a week | 362 | 11.18 |
|  | At least once a week | 497 | 15.35 |

*“Table 1 continued”*

| Individual level variables | Categories | Weighted | |
| --- | --- | --- | --- |
|  |  | Frequency | Percentage |
| Chewing chat | No | 2,660 | 80.78 |
|  | Yes | 632 | 19.22 |
| Alcohol drinking | No | 2,562 | 77.83 |
|  | Yes | 730 | 22.17 |
| Family planning unmet need | No unmet need | 2,108 | 64.02 |
|  | Unmet for spacing | 507 | 15.41 |
|  | Unmet for limiting | 677 | 20.56 |
| Total number of births | Mean and SD =5.10±0.044 | 95% CI: (5.014-5.19) | |
| Age at first birth | 11-14 years old | 228 | 6.92 |
|  | 15-19 years old | 2,011 | 61.08 |
|  | 20-24 years old | 1,053 | 32.00 |
| Ever had terminated pregnancy? | No | 2,925 | 88.84 |
|  | Yes | 367 | 11.16 |
| Sex of household | Male | 2,912 | 88.46 |
|  | Female | 380 | 11.54 |
| Relationship to Household | Head | 319 | 9.67 |
|  | Wife | 2,838 | 82.20 |
|  | Others* | 136 | 4.13 |
| Total household member | Mean and SD 6.28±0.038 | 95% CI:(6.21-6.36) | |
| Children under five at home | No | 443 | 13.47 |
|  | One | 1,353 | 41.11 |
|  | Two and above | 1,496 | 45.43 |
| Sons and daughters at home | No | 830 | 25.20 |
|  | Yes | 2,463 | 74.80 |
| Sons and daughters elsewhere | No | 2,258 | 68.57 |
|  | Yes | 1,034 | 31.43 |
| Sons and daughters died | No | 1,756 | 53.32 |
|  | Yes | 1,536 | 46.68 |
| wealth index | Poor | 1,645 | 49.96 |
|  | Middle | 644 | 19.55 |
|  | Rich | 1,004 | 30.49 |

*Others*= mother, sister, daughter, and others relatives and non-relatives a=Weighted samples*

Table 2: Community-level characteristics of pregnant women from Ethiopian DHS 2016 in Ethiopia (N^a^ =3,292)

| Community (cluster) level variables | Variable categories | Weighted | |
| --- | --- | --- | --- |
|  |  | Frequency | Percent |
| Place of residence | Urban | 218 | 6.62 |
|  | Rural | 3,074 | 93.38 |
| Region | Agrarian region | 2,946 | 89.47 |
|  | Emerging region | 305 | 9.26 |
|  | Metropolitan cities | 42 | 1.27 |
| Community illiteracy | Lower | 685 | 20.82 |
|  | Higher | 2,607 | 79.18 |
| Community poverty | Lower | 1,501 | 45.59 |
|  | Higher | 1,791 | 54.41 |
| Community-women empowerment | Lower | 1,681 | 51.06 |
|  | Higher | 1,611 | 48.94 |
| Community Media unexposed | Lower | 834 | 25.35 |
|  | Higher | 2,458 | 74.65 |
| Community unmet need for family planning | Lower | 2,108 | 64.02 |
|  | Higher | 1,184 | 35.98 |

Table 3: Random effects analysis for advanced-age pregnancy in Ethiopia based on Ethiopia Demographic Health Survey 2016.

| **Random effects** | **Null** | **Model I** | **Model II** | **Model III** |
| --- | --- | --- | --- | --- |
| Community variance | 4.82 | 4.03 | 4.28 | 3.12 |
| ICC % | 59.43 | 55.05 | 56.54 | 48.67 |
| PCV | Ref. | 16.39 | 11.20 | 35.27 |
| MOR | 2.09 | 1.91 | 1.97 | 1.67 |
| **Model fitness** | | | | |
| Akaike’s Information Criteria | 2548.91 | 1314.82 | 2484.43 | 1458.73 |
| Bayesian Information Criteria | 2561.08 | 1460.80 | 2533.09 | 1276.26 |
| Log-likelihood (LL) | -1272.46 | -633.41 | -1234.22 | -608.13 |

Table 4: Multivariate multilevel Logistic Regression Analysis for determinants of AAP from Ethiopian DHS 2016 in Ethiopia;

| Individual/household level | Advanced age pregnancy (weighted sample size) | | | | | |
| --- | --- | --- | --- | --- | --- | --- |
|  | Yes | No | Null | Model I | Model II | Model III |
| Religion | | | | | | |
| Muslim | 447 | 1,086 | _____ |  | _____ |  |
| Orthodox | 371 | 590 | _____ | 0.33(0.16-1.67) | _____ | 0.19(0.09-1.42)* |
| Protestant | 304 | 492 | _____ | 1.37(0.77-2.42) | _____ | 0.90(0.48-1.68) |
| Age at first birth (continuous) | | | _____ | *4.27(1.82-10.3)* | _____ | 4.05(1.77-9.22)* |
| Age at first sex (continuous) | | | _____ | 1.20(1.12-1.29) | *_____* | *1.22(1.13-1.31)** |
| Mothers education level | | | | | | |
| No education | 876 | 1,513 | _____ |  |  |  |
| Primary | 219 | 566 | _____ | 2.03(1.27-3.25) | _____ | *2.72(1.55-4.76)** |
| 2^ndary^  and above | 28 | 90 | _____ | 2.0(0.76-5.27) | _____ | *5.60(1.77-17.7)** |
| Women Occupation | | | | | | |
| Have no work | 568 | 1,215 | _____ |  | *_____* |  |
| Have work | 555 | 954 | _____ | 0.84(0.57-1.23) | *_____* | *0.93(0.62-1.40)* |
| Listening Radio or watching Television | | | | | | |
| Not at all | 772 | 1,519 | _____ |  | _____ |  |
| <one a week | 200 | 310 | _____ | 1.34(0.77-2.33) | _____ | *1.28(0.64-2.54)* |
| Once a week | 151 | 341 | _____ | 1.27(0.71-2.29) | _____ | *1.01(0.47-2.15)* |
| Drinking alcohol | | | | | | |
| No | 822 | 1,739 | _____ |  | _____ |  |
| Yes | 300 | 430 | _____ | 9.67(4.88-19.2) | _____ | *11.8(5.71-24.42)** |
| Wealth index | | | | | | |
| Poor | 483 | 1,162 | _____ |  |  |  |
| Middle | 253 | 391 | _____ | 3.25(1.98-5.34) | _____ | 1.59(0.85-2.97) |
| Rich | 387 | 617 | _____ | 2.99(1.78-5.03) | _____ | 1.42(0.72-2.79) |
| Parity | | | _____ | 2.80(2.39-3.29) |  | *3.22(2.69-3.84)** |
| Lists of household members | | | _____ | 1.30(1.13-1.48) |  | *1.22(1.05-1.41)** |
| Family planning unmet need | | | | | | |
| met need | 710 | 1,397 | _____ |  | _____ |  |
| Spacing Unmet | 271 | 236 | _____ | 1.64(1.02-2.63) | _____ | *4.79(2.63-8.74)** |
| Limiting Unmet | 141 | 536 | _____ | 1.05(0.67-1.67) | _____ | *1.64(0.95-2.84)* |
| Ever had a pregnancy termination | | | | | | |
| No | 980 | 1,945 | _____ |  | _____ |  |
| Yes | 143 | 224 | _____ | 0.69(0.39-1.23) | _____ | *0.66(0.36-1.22)* |
| Number of under-five at home | | | ______ | 0.27(0.21-0.35) | *_____* | *0.26(0.20-0.35)* |

“Table 1 continued”

| Advanced age pregnancy (weighted sample size) | | | | | | |
| --- | --- | --- | --- | --- | --- | --- |
| Variables | Yes | No | Null | Model I | Model II | Model III |
| Sons/ Daughters at home | | | | | | |
| No | 124 | 705 | _____ |  | _____ |  |
| Yes | 999 | 1,464 | _____ | 1.47(0.87-2.49) | _____ | 1.15(0.65-2.02) |
| Son/daughter elsewhere | | | | | | |
| No | 528 | 1,729 | _____ |  | _____ |  |
| Yes | 594 | 440 | _____ | 1.99(1.31-3.02) | _____ | 1.89(1.22-2.94)* |
| Sons/daughters died | | | | | | |
| No | 449 | 1,306 | _____ |  | _____ |  |
| Yes | 674 | 863 | _____ | 1.04(0.68-1.57) | _____ | 0.77(0.49-1.19) |
| **Community level variables** | | | | | | |
| Region | | | | | | |
| Agrarian | 1,039 | 1,907 | _____ | _____ |  |  |
| Emerging | 79 | 226 | _____ | _____ | 0.81(0.26-2.51) | 0.29(0.14-1.59) |
| Metropolitans | 5 | 37 | _____ | _____ | 0.20(0.028-1.38) | 0.10(0.03-0.38)* |
| community illiteracy | | | | | | |
| Lower | 212 | 474 | _____ | _____ |  |  |
| Higher | 911 | 1,695 | _____ | _____ | 9.18(2.83-29.76) | 1.88(0.89-3.98) |
| Community poverty | | | | | | |
| Lower | 601 | 900 | _____ | _____ |  |  |
| Higher | 522 | 1,269 | _____ | _____ | 6.25(2.13-18.39) | 2.37(1.16-4.85)* |
| Media exposure | | | | | | |
| Lower | 784 | 1,520 | _____ | _____ |  |  |
| Higher | 339 | 650 | _____ | _____ | 0.90(0.30-2.68) | 0.70(0.33-1.50) |
| Community unmet need | | | | | | |
| Lower | 710 | 1,397 | _____ | _____ |  |  |
| Higher | 413 | 772 | _____ | _____ | 1.87(0.67-5.24) | 5.19(2.72-9.92)* |
