# Supplementary material for: Determinants of advanced age pregnancy in Ethiopian; multi-level analysis of Ethiopian demographic health survey 2016
Source: PLoS One. 2024 Jun 25;19(6):e0304954. doi: 10.1371/journal.pone.0304954 (PMC11198901; doi:10.1371/journal.pone.0304954)
Supplement: Attachment — Submitted filename: Review.docx [file pone.0304954.s004.docx]

The manuscript entitled " Individual and community level Determinants of Advanced Age

Pregnancy in Ethiopia, Multi-Level Analysis of National Data 2016 " has been reviewed. The authors have studied the individual and community-level determinants of advanced maternal age pregnancy in Ethiopia. It seems that additional explanations are needed to be given in the manuscript to increase its quality. I have some recommendations to improve the quality of the article as follow.

**Method**

In the data collection section, it is not clear in what period of time the data was collected. If the data is for childbirth in 2016, when were the other variables collected? Please clearly state the data collection date.

In the sample size measurement section, explain why 645 samples were taken and how many were selected in each cluster.

Explain more about the weighting and how it was.

In the eligibility criteria section, why were visitors who stayed at home the night before the survey included?

**Results and discussion**

In the results section, the tables must be given inside the text of the site. Also, below the tables, it should be specified what variables are included in the first model and how it differs from the third model.

Finally, it should be determined which model's efficiency and goodness of fit are better and which model's results should be used?

The results of Multilevel regression analysis and should be stated separately in a paragraph.
