# Supplementary material for: Determinants of advanced age pregnancy in Ethiopian; multi-level analysis of Ethiopian demographic health survey 2016
Source: PLoS One. 2024 Jun 25;19(6):e0304954. doi: 10.1371/journal.pone.0304954 (PMC11198901; doi:10.1371/journal.pone.0304954)
Supplement: Attachment — Submitted filename: Response to reviewer.docx [file pone.0304954.s005.docx]

**“Responses to reviewer”**

**Responses to reviewer #1**

I. **Method**
1. In the data collection section, it is not clear in what period of time the data was collected. If the data is for childbirth in 2016, when were the other variables collected? Please clearly state the data collection date.

**Response; Thank you!**

- The data collection of Ethiopian DHS 2016 was conducted from January 18 to June 27, 2016.
- Since Ethiopian DHS is a cross-sectional survey in nature all the data were collected at the same time. But the data was compiled into house hold, man, woman, birth history, and under-five records at the individual level. From these records, we use birth records (BR) for this research, because this record contains all the variables, we want to test in the analysis depending on our literature.

2. In the sample size measurement section, explain why 645 samples were taken and how many were selected in each cluster.

**Response; Thank you!**

- The sampling size determinations for DHS data sets where mainly two stages depending on the recently conducted census in the country before the DHS data collection. It is also true for Ethiopia.
- Stratification is the process by which the sampling frame is divided into subgroups or strata that are as homogeneous as possible using certain criteria like regions, and place of residence (rural and urban). The first stage is the so-called enumeration areas (EAs) considered as primary sampling unit (PSU). The primary sampling unit forms the survey cluster. In the second stage, a complete household listing is conducted in each of the selected clusters. Following the listing of the households a fixed number of households is selected by equal probability systematic sampling in the selected cluster.
- In each enumeration area DHS took a fixed number of households around 25-30 (on average 28 households in each stratum), which determines the number of clusters required. In this research, from the Ethiopian recent census, there were 645 enumeration areas (PSU) (202 EAs in urban areas and 443 EAs in rural areas) selected at the first sage and then 28 households from each cluster. 16,650 households, 15,683 female respondents, and 12,688 male respondents were included at last.

3. Explain more about the weighting and how it was.

**Response; Thank you!**

- Sampling weights are adjustment factors applied to each case in tabulations to adjust for differences in the probability of selection and interview between cases in a sample, due to either design or happenstance. The EDHS samples were not self-weighted due to the need for data for specific regions or areas of the country that often need to be over-sampled. Thus, we applied sample weights to the data when we computed the analyses.

4. In the eligibility criteria section, why were visitors who stayed at home the night before the survey included?

**Response; Thank you!**

- Since it is secondary data from DHS, the survey follows their manual to conduct it. Standing from that the eligibility criteria for women to be interviewed in the DHS. After selection of the community, the next to be selected is the household. All women usually age 15-49 who stayed in the household the night before the survey.

II. Result and Discussion
1. In the results section, the tables must be given inside the text of the site. Also, below the tables, it should be specified what variables are included

**Response;** Thank you!!!

- Corrected!!!

2. In the first model and how it differs from the third model. Finally, it should be determined which model’s efficiency and goodness of fit are better and which model’s results should be used.

**Response; Thank you**

- The first model it is actually null/empty model only we test the dependent variables?
- The second model tests the outcome variables and the individual level factors (Model-I)
- The third model tests the outcome variables and the community level factors (Model-II)
- The fourth model tests the outcome variables with both individual and the community level factors (Model-III)
- Model fitness was checked by Loglikelihood and Akaike and Bayesian information criteria. We took the smallest figure indicating the model is nicely fitting in the last model (model-III).

3. The results of Multilevel regression analysis should be stated separately in a paragraph.

**Response; Thank you**

- We have Corrected! We state into two separate tables
  - Table 3 is about the random effects and model comparison, before the table we tried to describe what the table mean.
  - Table 4 is about multivariable multilevel regression analysis results and we are described the key and significant predictor variables

The manuscript needs language editing.

**Response;** Thank you for your genuine comments/concerns and questions as well.

- We are trying to solve language issues by online soft-wares like Grammarly and manually with repetitive reading and paraphrasing.

**Reviewer #2:**

Title: It is better to express the title with a more measurable format.

**Response;** thank you; the title is modified as “determinants of advanced age pregnancy in Ethiopia; multi-level analysis of Ethiopian demographic health survey 2016
Abstract: Please convey the objective of the study in a more measurable format. It is not necessary to show the analysis method in the objective of the study.

**Response**; Thank you, there is no objective section in the Abstract, but there is method section; describes source of data, sample size and modes of analysis and the like.
Keywords: OK

**Response**; Thank you
Introduction (background): there are a number of abbreviations which are not clear for what they are standing for, in contrast to their popularity (e.g.; AMA). I think it is good to express the reason you are using the DHS 2016 (seems to be an old one).

**Response**; Thank you for your genuine concern,

- we have rewrite in formal (long term) for all abbreviations (corrected) and the reason we are using DHS 2016, it is the last major DHS in Ethiopia. Off course we have Mini Ethiopia DHS 2019, but it covers a small number of population and variables.

**Material and method: Please note to the following issues in this section:**
- Please define the proposed independent variables in more detail so that the measurement method will be clear.
- Please define how community level variables were collected and estimated. On the other hand, I could not understand why residence is considered as a community-level variable. I think it is also important to show how community-level variables were assigned to each individual. You have tried to explain the issue in the “Multilevel regression analysis” section, however, I think more definition is necessary. Maybe explaining the sampling method of the DHS especially the clusters, will suffice, especially in terms of the data and multilevel nature of it.
- There are a number of variables which are not defined in this section (e.g.; chewing chat or alcohol drinking). On the other hand, it is necessary to show the reliability of the responses as well.
- Please show the software used for the analysis of the data, together with the statistical significance level of the study.

**Response**; thank you for your genuine concern on methods and materials section

1. The proposed independent variables were well described in the operational definitions and variable sections of the methodology of the research.
2. Concerning community level variables; since DHS is cross-sectional survey all the variables were collected at the same time. There is no follow-up and the like. The community level variables were estimated from the individual variable (response) by principal covariate analysis (PCA).it is based on the median value of the individual response in the DHS data depend on the interest of our research. From DHS itself residence and regions are directly measures the community level variables that the particular participant’s cluster (enumeration) area actually existed. The responses for the residents of that particular participant are urban or rural and it directly indicates household location. Contextual regions also one of the community level factors directly used from the Ethiopian DHS.
3. Each community level variables are assigned to the individual variable through their cluster. We use a variable called individual cluster variable. for house-hold there is a corresponding cluster in the selected house-holds. There are different cluster numbers V001, hhv001, and mv001for woman, house-hold and man respectively. Therefore, the cluster is predefined in recent national censes before this survey. The number of house-holds in each cluster also in average known, 28 house-holds were taken from each selected cluster and then eligible woman and man interviewed.
4. The Ethiopian demographic health surveys follow a two-stage stratified sampling design like that of most DHSs, where Primary Sampling Units (PSU), also referred to as clusters, are selected in the first stage following a probability proportional to size (PPS) selection. The samples are typically stratified by regions and urban/rural residence.
   a. In the first stage of sampling, PSUs are selected according to the allocation of the PSU sample over the sample strata. After the selection of the PSUs, a household listing operation is conducted in every selected PSU and a complete list of households is constructed. This clusters (PSU) are enumeration areas which is from a recent country census used as a sampling frame for the first-stage selection. The EAs are selected as PSUs or clusters. An EA is a geographical area that serves as a counting unit for the population census, and is usually a city block in urban areas or a village in rural areas with 200-300 households.
   b. In the second stage, a fixed number of households is selected from each cluster (PSU). From the selected households, all women age 15–49 who slept in the household the night before the survey is eligible to complete a questionnaire designed for women. In addition, in all households or in a subsample, men of reproductive age (typically 15–59) are eligible to complete a questionnaire designed for men.
5. Variables are defined in the independent variable sections. The definition includes the way we are operationalized for analysis, but some how there are differences in the descriptive statistics and in the multi-level analysis, because presence of outliers, insignificant number (percentage) of variable category affects the concavity(convergence) of the analysis. In that case recategorization and rename we took place.
6. Variables like alcohol and chat were directly taken from the survey since their response yes/no. the reliability of the response is Under question? Therefore, we put this as a limitation in the study.
7. The software we used for this analysis was STATA 14. We can use commands (STATA commands) and menu. We declare the statistical significance level by Adjusted Odd Ratio (AOR) with 95% confidence interval and p-value ≤0.05.

Results: It is necessary to show the proposed characteristics in each group of advanced age pregnancy and the other one separately. Showing the estimated effect measure is not enough with this regard.

**Response**; **Thank you!!! It is corrected as stated below**

1. In our result section table 1 and 2 describes the characteristics of Advanced age pregnancy and their counter parts (non-Advanced age pregnancy) in terms of their sociodemographic, economic, behavioral and community level characteristics. In the last table currently (table 4) indicates the associations the outcome of interest and the predictor variables with adjusted odd ratio and P-value less than 0.05 are significant level to declare the predictors determining or not. On this regard we put only the odd ratio for space matter in the table, and *(asterisk) on significantly important variables, and we described under the table for each p-value.

**Discussion: OK; however, a number of revisions may be required following revising of the previous sections.**

Response; thank you!!!

1. We are trying to reshuffle/paraphrase depend on the comments given in the methods and results sections.
   References: OK

- **Thank you for your time and genuine constructive comments!!!**

**Reviewer #3:**

The study in Ethiopia examined factors contributing to advanced maternal age pregnancies utilizing data from the 2016 Ethiopian Demographic and Health Survey. It involved analyzing 3,292 weighted samples of pregnant women. Using a multilevel logistic regression model, the research identified that increased maternal age at first birth, lower levels of maternal education, history of alcohol consumption, greater parity (number of children), unmet needs for family planning, having children residing outside the home, higher community poverty levels, and unmet community needs for family planning were associated with an increased likelihood of advanced-age pregnancies. Conversely, being located in an emerging region or living in urban centers like metropolitan cities showed negative associations with advanced-age pregnancies.

However, there are several questions in this study.

1. In statistical analysis, "bivariate" usually refers to examining the relationship between two variables at the same time. I did not understand what the authors meant in this study.

**Response; Thank you very much!!!**

- Bivariate analysis in this case is used to test the relationship between the outcome variables and each predicting variable turn by turn in order to determine the variable is important for the next analysis depend on the p-value we set (p-value<0.25). we analyzed the crude associations of individual and community factors with advanced-age pregnancy by considering the sample design of the Ethiopian demographic health survey using the cluster variable. Both bi-variable and multi-variable multilevel logistic regression were performed to assess the independent effect of the individual and community level variables on advanced maternal age pregnancy. Independent variables with p-values less than 0.25 in the bi-variable multi-level analysis were entered into the multivariable multi-level logistic regression analysis.

2. The statement "mothers are nested within households, and households are nested within clusters" suggests a hierarchical structure where individuals (mothers) are grouped within households, which in turn are grouped within larger clusters or communities. While a three-level model could have been more appropriate to account for this nested structure, the authors opted for a two-level model in this study. This decision may have overlooked the potential influence of the family unit on advanced maternal age pregnancies.???

**Response: thank you very much**

- Since the demographic health survey are two stages, the community (primary sampling unit), and the house-holds. From the selected households, all women age 15–49 who slept in the household the night before the survey is eligible to complete a questionnaire designed for women. In addition, in all households or in a subsample, men of reproductive age (typically 15–59) are eligible to complete a questionnaire designed for men.

3. Interview date can indeed be considered a confounder in the models if it is associated with both the exposure (factors contributing to advanced maternal age pregnancies) and the outcome (advanced-age pregnancies). If interview date is related to any of the variables being studied and could potentially influence the results, it should be accounted for in the analysis to ensure accurate and unbiased findings.

Response; Thank you!!!

- Interview date we don’t know it is or not because of difficulty for analysis. The problem we face on this regard is we don’t know the interview date of each community (cluster). On the interview date the exact date (month) doesn’t specify in the survey data rather the put as numbers like 1301,1302…and the like.

4. **In the Combined model:**
a. The formula does not specify the meaning of variables i and j.

**Response: thank you!**

- I and j in the formula specifies the i^th^ pregnant woman in the j^th^ community (cluster)

b. Variables i and j are not clearly labeled under the index.

**Response: Thank you**

- We added to the legend part after the formula and highlighted in the manuscript.

c. Uncertainty exists regarding whether β_0 represents the width from the overall intercept.

**Response: Thank you**

- β0; it is the intercept/slope of the outcome variable is equal to in the absence of variable.
- In this case
  - β_0 denotes the log odds of the intercept when in the absence of the predictor variable/s.

d. suggests the authors to use distinct variables for individual and social independent variables such as X and w.

**Response: thank you!**

- of course, it is possible use different symbols for individual and community level variable, but it has no as such significance difference unless to simplify for the readers. so we change from x1, x2…., xn to X and Z for each individual and community level respectively.

$$\log\left[ \frac{\pi ij}{1-\pi ij} \right]=\beta o+\beta1Xij+\beta2Zij+\cup j$$

b. The presence of random errors (u_j) raises questions about the location of random effects within the model.

**Response: thank you**

- Uj showed the random effect (effect of the community on advanced age pregnancy for the jth community (cluster) the woman belongs to.
- From the Above equation there are two categories, variables (predictors) with Beta coefficients are called fixed effects and the remaining uj is called random effects.
- In multilevel analyses, we incorporated a random effect at the cluster level to account for the clustering of individuals within communities.

c. The distribution of u_j remains unknown, adding to the uncertainty surrounding the model's components.

**Response: thank you**

- random effects (uj) are uncorrelated with the fixed effects (coefficients), and simply show uncertainty due to the community.
- There is also unexplained variation around the expected value.

5. The authors' use of "log distribution" was unclear. It should refer to a standard logistic distribution with variance.

**Response: thank you**

- We use multi-level binary logistic regression, since the outcome of interest was binary (yes/no). due to hierarchical data types, we use multilevel logistic regression at the cluster level. This is different from the standard logistic regression.

6. The statement regarding the residual variance of women within a cluster in log distribution should be clarified to involve a power term, not just a constant.

**Response: thank you**

- The residual variance is the difference between the observed values and the predicted values in a regression model. So, it measures the amount of variability that is not explained by the model and key indicator of how well the model fits the data.

7. Please provide the PCV formula.

**Response; thank you!**

- Proportional change of variance is calculated as $PCV=\frac{Va-Vb}{\mathrm{Va}}$
- Where Va is the variance in the null model and Vb is the variance in the consecutive models

8. It is suggested to calculate MOR (Mean Odds Ratio) and add it to Table 3.

**Response; thank you**!

- Median odd ratio the heterogeneity between the higher and lower risks of being advanced age pregnancy. So, it is calculated as:

$MOR=(exp\sqrt{2\times0.6745\times v1})\approx exp(0.95\sqrt{v1})$. V1 is the area-level variance, and 0.6745 is the 75^th^ percentile of the cumulative distribution function of the normal distribution with 0 mean and 1 variance.

9. Please add the ICCcom formula and guidance on its interpretation to decide on the analysis method.

**Response; thank you!**

- I think you mean say ICCcom; -community-level intraclass correlation. We calculated intraclass correlation for each model and the result was provided Table 3 of this manuscript. It is calculated as follows; $ICC=\frac{\mathrm{Va}}{Va+\pi2/3}$ where π^2^/3=3.29 make
- $ICC=\frac{\mathrm{Va}}{Va+3.29}$ , Va is the estimated variance in each model.

10. STATA software lacks multilevel models, prompting a discussion on the statistical analysis methods or software packages utilized in the study.

**Response; thank you!**

- We used STATA version 14, with commands (melogit dependent variable (independent variables) [weight] ||cluster number: This is the command we use for multilevel Analysis. We can also run multilevel analysis with the STATA menu as follows. Menu>statistics>multilevel>logistic regression.

11. Models referenced in Table 3 require further elaboration and description.

**Response: thank you!**

- We elaborate on it. We reconstruct the table in two separate parts
  - Table 3; shows the random effect and model fitness comparison, before the table we have described what the table means. especially the random effect values and model fitness and
  - Table 4 is about multivariate multi-level regression analysis and we tried to elaborate on key findings in the table
